# Microwave-assisted synthesis of trimethylolpropane triester (bio-lubricant) from camelina oil

**DOI:** 10.1038/s41598-022-16209-z

**Published:** 2022-07-13

**Authors:** Kian Rokni, Mostafa Mostafaei, Masoud Dehghani-Soufi, Danial Kahrizi

**Affiliations:** 1 Department of Biosystems Engineering, Faculty of Agriculture, Razi University, Kermanshah, Iran; 2 Department of Agrotechnology, College of Aburaihan, University of Tehran, Tehran, Iran; 3 Department of Agronomy and Plant Breeding, Faculty of Agriculture Science and Technology, Razi University, Kermanshah, Iran

**Keywords:** Energy science and technology, Renewable energy

## Abstract

Vegetable oils, whose hydrocarbon structure is very similar to that of petroleum products, are ideal renewable and sustainable alternatives to petroleum lubricants. Bio-lubricants are commonly synthesized by modifying the chemical structure of vegetable oils. In this study, microwave irradiation was applied to intensify the mass-transfer-limited transesterification reaction to produce trimethylolpropane triester (bio-lubricant) from camelina oil as a promising local energy crop. A rotatable RSM-BBD method was applied to find the optimal levels of experimental factors, namely reaction time (67.8 min), the catalyst concentration (1.4 wt%) and the molar ratio (3.5). In these optimal levels, the reaction yield of 94.3% was obtained with desirability of 0.975. The quadratic statistical model with a determination coefficient of 97.97%, a standard deviation of 0.91 and a variation coefficient of 1% was suggested as the most appropriate model by Design-Expert software. Finally, the physicochemical properties of the purified product were in accordance with the requirements of the ISO-VG22 base oil standard.

## Introduction

As world population projections soar, there are rising concerns about basic human needs, such as water, raw materials and energy. The best-principled way to meet these challenges is to apply sustainable technologies. The concept of sustainability implies that industries should take serious steps to comply with the principles of circular economy and the United Nations sustainable development goals (SDGs). Excessive use of fossil fuels and crude oil, in addition to environmental issues, has also raised serious concerns about the energy supply as well as the other petroleum derivatives in the future. Therefore, many countries and international organizations have made strict policies to develop advanced bio-based products^1^.

Lubricants are one of the most important added-value products derived from petroleum and their global consumption is constantly increasing with the increasing industrial development of countries. Lubricants are essential for almost all aspects of modern machinery. As the name implies, lubricants are substances used to lubricate surfaces in mutual contact to facilitate the movement of components and reduce friction and wear^2^. Lubricants can be found in a variety forms including gases, liquid products (mineral oils, animal and plant oils, derivatives of fatty acids, synthetic oils, and water-based fluids), grease^3^. Reducing energy losses by reducing friction and heat loss between moving parts in mechanical systems is the main use of lubricants. More than 85% of lubricants are produced from crude oil and 5–10 million tons of their toxic wastes enter the environment annually through accidental spillage, refining process, and irresponsible disposal in water, soil or sewage. About 50–60% of lubricant waste is in direct contact with soil and water, and one kilogram of oil lubricant can contaminate one million liters of drinking water^4^. This poses a great threat to human, animal and plant life and has led to the increasing development of environmentally friendly lubricants, especially in applications in the agricultural and marine industries^5^.

Compared with mineral lubricants, which account for more than 85% of the lubricant market share, bio-based lubricants have very desirable features such as lower price, higher viscosity index, better lubricity and excellent fatigue reduction in parts (twice as much as mineral lubricants), higher load-carrying capacity, lower volatility and better compatibility with lubricant additives^6^. Bio-lubricants based on saturated fatty acids often have high oxidative stability and flash point. This is due to the properties of saturated fatty acids with high oxidative stability and flash point led to produce biolubricant with good lubrication^3^.

Trimethylolpropane triester (TMPTE) production from different types of oil-bearing biomass resources through transesterification reaction has been commonly investigated in many research works in the last decade^7–11^. Transesterification reaction of TMPTE synthesis is a reversible time-consuming process, which in its progress, is highly dependent on the rate of mass and heat transfer. Therefore, some process intensification techniques are used, such as hydrodynamic cavitation^12–15^ novel co-solvents^16^ and ultrasonic irradiation^17,18^. Also, Aziz et al*.*, in a review paper recommended the use of microwave technology in intensifying bio-lubricant production^19^.

The heating process in conventional transesterification reaction involves forced conduction and convection based on direct molecular collisions. Indeed, the temperature gradient between the internal reactor wall and the reactants as well as the conductivity of the reactor wall play key roles in the conventional heating process. Microwave radiation is unique due to the fact that the heating mechanism is generally controlled by direct radiation to a molecular level, based on the motion of charged protons and electrons. Therefore, it can reduce processing time, save energy and improve production yield, which is attributed to the intense heating at molecular level^20^. The microwave is part of the electromagnetic spectrum with a wavelength of 1 m to 1 mm, which corresponds to a frequency range of 300 MHz to 300 GHz. Common frequencies for home and industrial heatings are 915 MHz and 2.45 GHz. The two main mechanisms that produce heat are based on the polarization of the material and ion heating. The polarity and ionic properties of materials are affected by rapid changes in the electromagnetic field. In addition, microwave heating affects the internal energy and reduces the activation energy of the reaction, which is similar to the catalytic effect in accelerating the reaction. This friction and molecular collision improve the intermolecular mixing of the reactants^21^.

**Figure 1:**
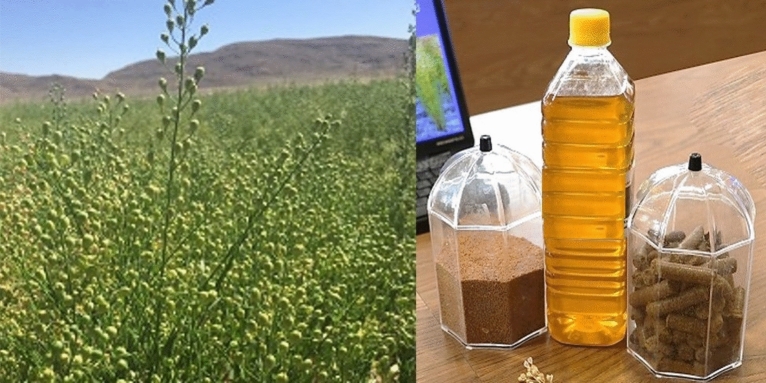
Camelina (Soheil cultivar): plant, seed, oil and oil cake.

The *Camelina sativa* is a member of the *Brassicaceae* family (Fig. 1) and has been shown in various experiments to have much lower water requirements and greater resistance to spring frosts than other oil plants, especially canola. Camellia oil is used in both edible and industrial types. The percentage of Camelina seed oil has been reported to be 29 to 41%^22^. Results of GC-Mass showed that Camellina oil is composed of palmitic acid (6.45%), stearic acid (2.56%), oleic acid (16.41%), eicosenoic acid (14.09%), erucic acid (3.21%), linoleic acid (21.03%) and linolenic acid (29.70%). “Soheil cultivar” is a dryland crop that requires less agricultural inputs than its competitors and is capable of growing in most regions of Iran. This cultivar has been released, propagated and cultivated for the first time at Biston Shafa Co., Razi University, Kermanshah, Iran^23–25^.

The *Camelina sativa* is suitable for temperate climates and poor soils that can withstand drought and heat. It grows rapidly and has a short life of 85 to 100 days. The higher the amount of unsaturated fatty acids, the lower the viscosity at room temperature and the stronger the frictional and abrasive properties of the oil. Also, oils with a high percentage of monounsaturated fatty acids (such as oleic acid) have a low coefficient of friction and wear rate and ensure greater oxidation stability at high and low temperatures^26,27^.

A review of the gap in the literature showed that microwave technology has not yet been introduced to intensify the bio-lubricant production process. This work addresses the intensification of the transesterification process of TMPTE production from Camelina oil (Soheil cultivar) as an indigenous cost-effective energy crop using microwave technology. The effect of some parameters in microwave-assisted bio-lubricant production has not yet been clarified; as the molar ratio of methyl ester to TMP, the weight ratio of catalyst to methyl ester and reaction time. Finally, the optimal parameters of the process are determined by Response Surface Methodology and Box-Behnken Design (RSM-BBD). Theis work seeks to bring new information and method to the domain of study for process intensification of bio-lubricant production. The general idea and purpose of this research is to prove the following hypotheses: The use of microwave method accelerates and improves the bio-lubricant production process and there is a direct relationship between microwave power and reaction time with the reaction efficiency of bio-lubricant production.

## Materials and methods

### Materials

The Soheil cultivar of Camelina oil was provided by Biston Shafa Co., Razi University. First, some important properties of oil such as acid number, free fatty acids (FFA) content, water content, saponification number and fatty acid profile were determined with the help of conventional chemical methods and gas chromatography (GC). The physicochemical properties of Camelina oil are tabulated in Table 1. we confirm that all methods applied on the Camelina plant were performed in accordance with the relevant institutional, national, and international guidelines and legislation.

**Table 1:** Physicochemical properties of Camelina oil (Soheil cultivar).

Property	Value	Standard
Oil content (wt%)	35	–
Molecular weight (g/mol)	899.24	–
Density at 15 °C (g/cm^3^)	0.910	ASTM D1298
Viscosity at 40 °C (mm^2^/s)	30.1	ASTM D445
Total acid number (mg KOH/g oil)	0.11	ASTM D664
Total base number (mg KOH/g oil)	187.16	ASTM D2896

Methanol with a purity of 99.6% was purchased from Dr. Mojallali Company, Iran. Trimethylolpropane (2-ethyl-2-(hydroxymethyl)-1,3-propanediol), potassium carbonate, and potassium hydroxide (all with analytical grade) were purchased from Merck. The materials and equipment used in this research were listed in Table 2.

**Table 2:** The materials and equipment used in this study.

Device	Manufacturer	Materials	Manufacturer
Microwave	Panasonic	Trimethylolpropane (TMP)	Merck
Vacuum pump	GAST	K_2_CO_3_	Merck
Stirrer (hot plate)	GENWAY	KOH	Merck
Oven	BINDER	Camelina oil	Razi Univ
Digital balance	Denver	CH_3_OH	Dr. Mojallali Co
DC armature (stirrer)	–	FTIR spectrometer	Bruker

### Equipment and experimental setup

The microwave oven used in this study was a Panasonic NN-ST34 with 25 L volume and 5 different power levels. Apart from light, loss of electrical power and other power consumptions, the device has a constant electromagnetic radiation power of 800 W and can generate five power levels by disconnecting and connecting the electromagnetic radiation in different cycles. The maximum total consumed power of the device was 1200 W (measured by a Lutron 6060 wattmeter). The Low mode state (irradiated power of 800 W) was selected as the most effective state for bio-lubricant production, otherwise, the material inside the reactor would overflow or the microwave device would shut down because of over power load.

**Figure 2:**
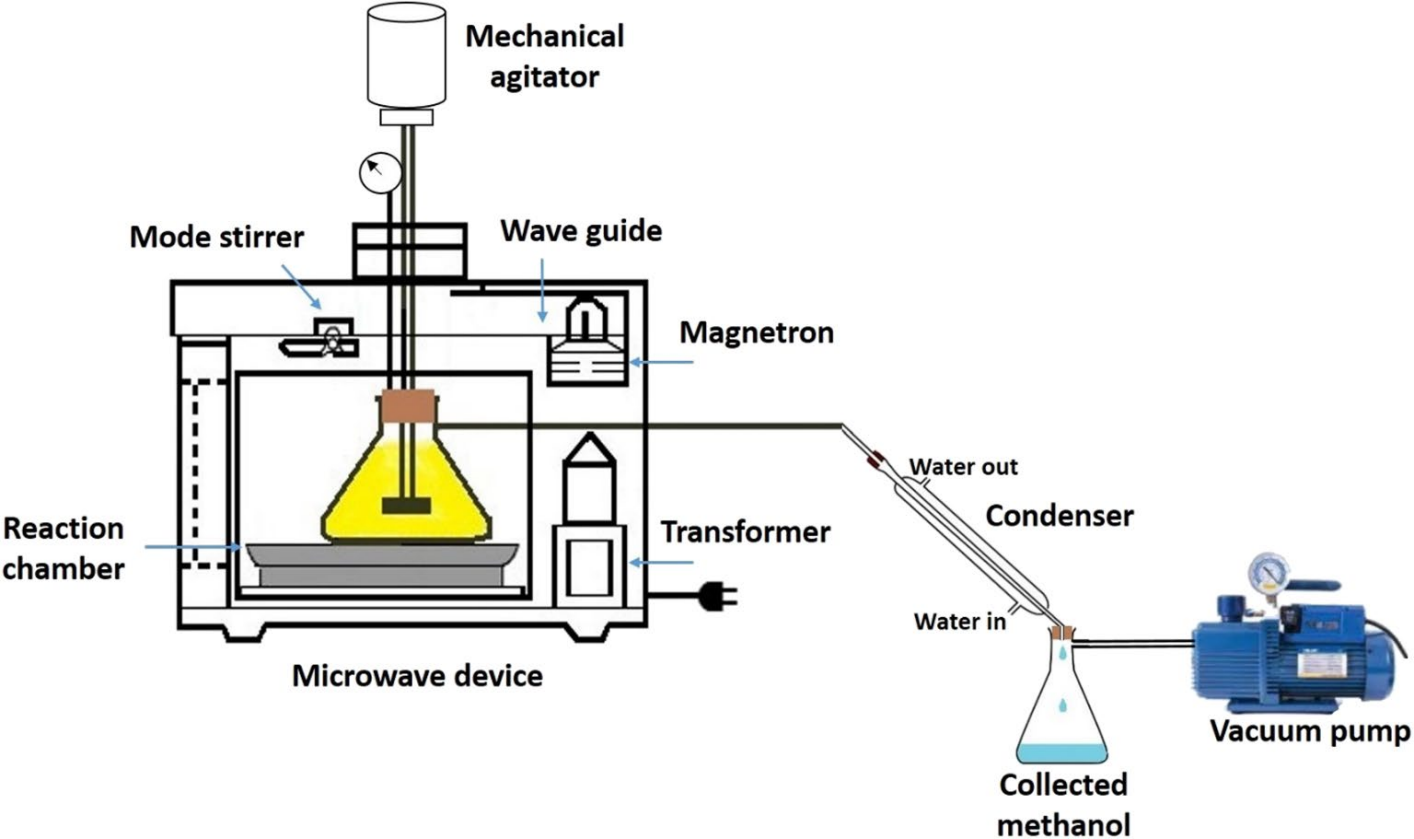
The experimental setup.

During each experiment, the microwave power was constantly on the “Low” mode. In this mode, the 800-W electromagnetic waves continuously irradiated the reactants for 5 s at intervals of 30 s (5 s ON and 30 s OFF)^28^. A flexible Teflon rod (T shape) attached to the armature shaft was used to stir the material inside the Erlenmeyer (250 cc). The rotational speed of the shaft was kept in the range of 450 rpm. The experimental setup is depicted in Fig. 2.

### TMPTE synthesis

**Figure 3:**
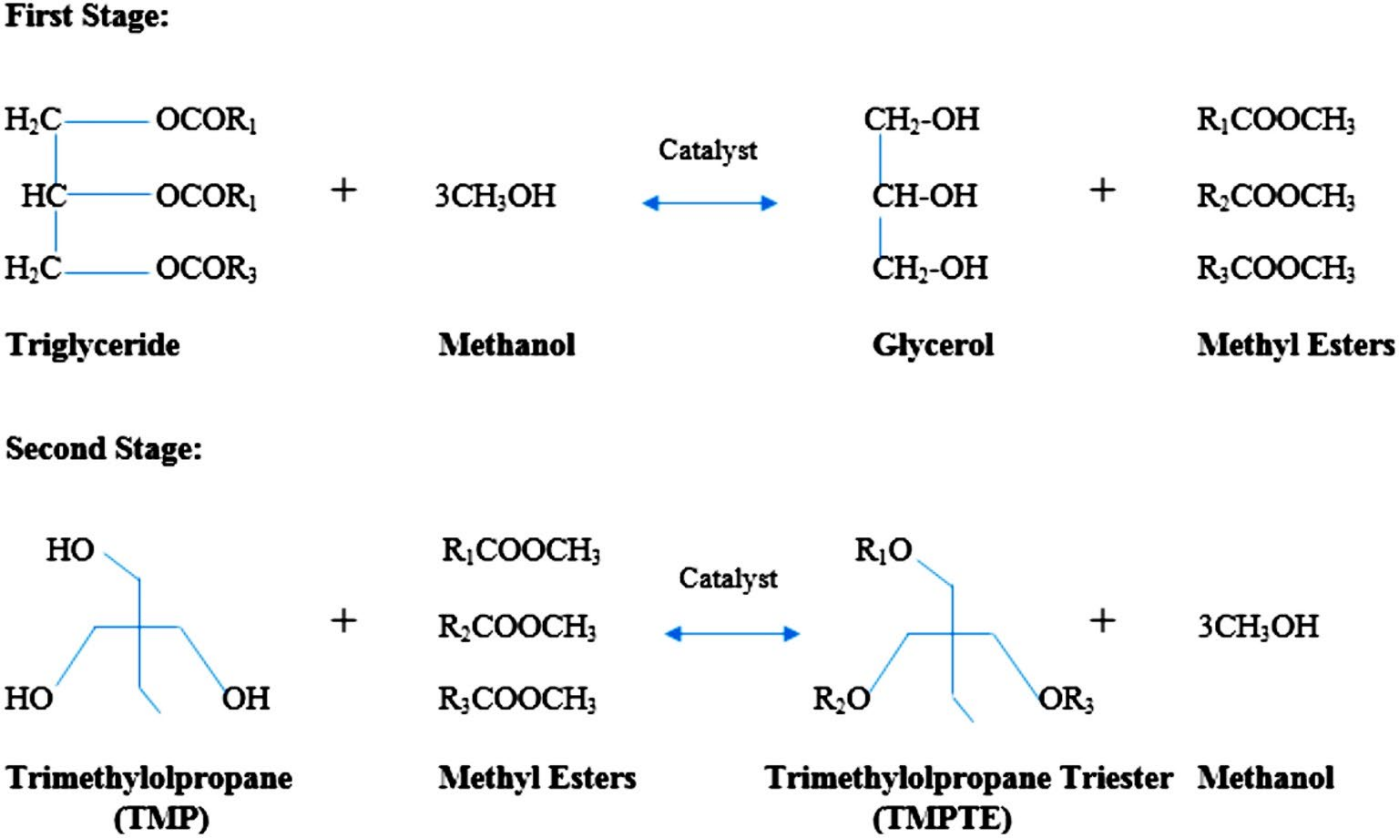
Stages of bio-lubricant production by transesterification method.

In the production process of TMP triester, Trimethylolpropane (TMP) polyhydric alcohol was used, which is a widely used polyol in the polymer industry. At this stage, potassium carbonate K_2_CO_3_ was used as a catalyst due to its insolubility in alcohol, availability, cheap price, heterogeneity, ease of separation from the reaction medium and reusability in the bio-lubricant production process. The transesterification reaction is based on mass transfer, which takes place in two stages of methyl ester and bio-lubricant production. In the first stage, the reaction to produce methyl ester, oil and methanol are two separated phases, and it is necessary to transfer the mass to form an emulsion from two solutions. During the mass transfer, the triglyceride molecule reacts with methanol to produce glycerol and methyl ester. In the second stage, in order to produce bio-lubricant to break down the steroid group in methyl ester, an alcoholic group of TMP is used to produce new esters based on vegetable oil (Fig. 3). In both stages, stirring and microwave irradiation were used to increase the contact between the liquid phases, the mass and heat transfer rate. A rod made of refractory Teflon attached to the armature shaft was used to agitate the reactants inside the reactor vessel. The armature was installed on the outer body of the microwave device and Teflon tape was used to seal between the armature body and the vessel. During all experiments, the microwave was set to Low mode and stirring was performed continuously.

### Experimental design

Several factors can be influential on the experiments that the response surface methodology (RSM) was used to avoid wasting time and cost. The effect of variable parameters was investigated and modeled using Design Expert 10 software and Box Behnken experimental design. In bio-lubricant production, factors such as time, catalyst load and the molar ratio were investigated (Table 3).

**Table 3:** Experiments carried out by Box Behnken design.

Run	Time (min)	Catalyst K_2_CO_3_ (wt%)	Molar ratio	Rection yield (%)
1	30	1.75	4	89.9
2	90	1.25	5	94.6
3	60	1.25	4	92.8
4	60	1.25	4	93.1
5	90	0.75	4	91.1
6	60	1.25	4	92.6
7	60	1.75	3	95.0
8	60	1.25	4	92.0
9	30	1.25	5	83.8
10	30	0.75	4	78.3
11	60	0.75	5	88.2
12	90	1.25	3	93.7
13	60	0.75	3	90.6
14	60	1.75	5	94.2
15	30	1.25	3	90.6
16	90	1.75	4	93.3
17	60	1.25	4	91.0

## Results and discussions

### Product analysis

**Figure 4:**
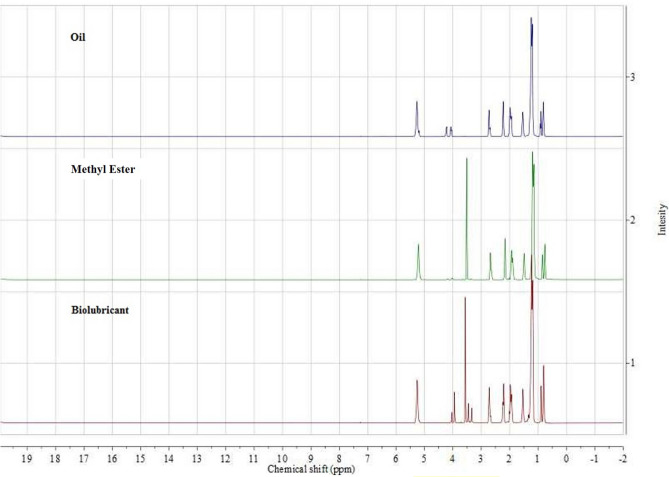
H-NMR spectrum curves for camelina oil, methyl ester and bio-lubricant.

A 500 MHz nuclear-hydrogen magnetic resonance spectroscopy (H-NMR) BRUKER, was used to determine the reaction efficiency. This spectroscopy relies on the magnetic resonance of the atomic nucleus and is used to identify the structure of organic compounds. For analysis, 2 ml of produced bio-lubricant with 0.1 ml of di-chloroform (CDCl_3_) was poured into a 5 ml tube and placed inside the device. The reaction yield of the reaction was calculated using Mestrenova 11.0.4 software and Eq. (1)^29^:

1 $$\mathrm{C\%}=\frac{1.02\mathrm{B}+\mathrm{A}}{1.52+\mathrm{A}}\times 100$$

which, A is the area under the peak with a chemical displacement of 4.02 ppm and B is the area under the peak with a chemical displacement of 4 ppm. The Box Behnken design used in this study and yield obtained by H-NMR are tabulated in Table 3. Also, Fig. 4 illustrates the H-NMR spectrum of camelina oil, methyl ester and bio-lubricant samples.

### FTIR analysis

**Figure 5:**
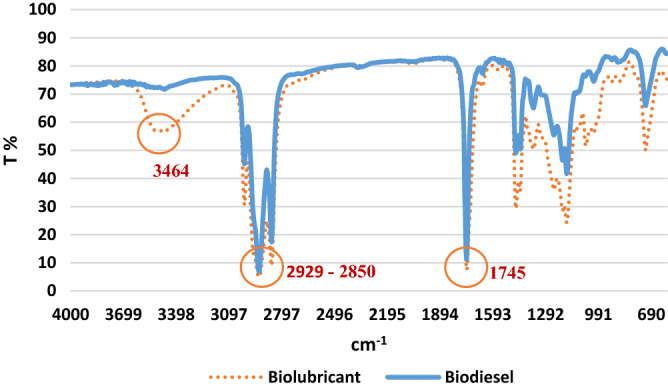
FTIR spectrum curves for methyl ester and bio-lubricant derived from camelina oil.

FTIR spectroscopy was used before evaluating and optimizing the results of transesterification reactions. As shown in Fig. 5, the FTIR spectra of the two samples of methyl ester and bio-lubricant from camelina oil have different peaks due to the presence of different functional groups. At the point 1745 cm^−1^, a significant peak is formed, which indicates the presence of carbonyl groups (C=O). Peaks between 2850 and 2929 cm^−1^ indicate the presence of methylene groups (CH_3_). The presence of a distinct peak at 3464 cm^−1^ indicates the tensile vibration of the –OH functional group in the bio-lubricant^30^.

### Statistical analysis

Based on the results of the analysis of variance (ANOVA), the quadratic polynomial equation was proposed by Design-Expert software as the most suitable regression equation in mathematical modeling for estimating the reaction efficiency of bio-lubricant production. This equation was obtained to estimate the bio-lubricant reaction yield based on the coded values of the independent variables as a relation with 97.97% coefficient of determination, 0.91 standard deviations and 1% coefficient of variation.

2 $$ \begin{aligned} {\text{Y}} & = 92.307 + 3.761*{\text{A}} + 3.029*{\text{B}} - 1.135*{\text{C}} \hfill \\ & \quad - 2.370*{\text{A}}*{\text{B}} + 1.899*{\text{A}}*{\text{C}} + 0.409*{\text{B}}*{\text{C}} \hfill \\ & \quad - 2.741*{\text{A}}^{2} - 1.413*{\text{B}}^{2} + 1.115*{\text{C}}^{2} \hfill \\ \end{aligned} $$

**Figure 6:**
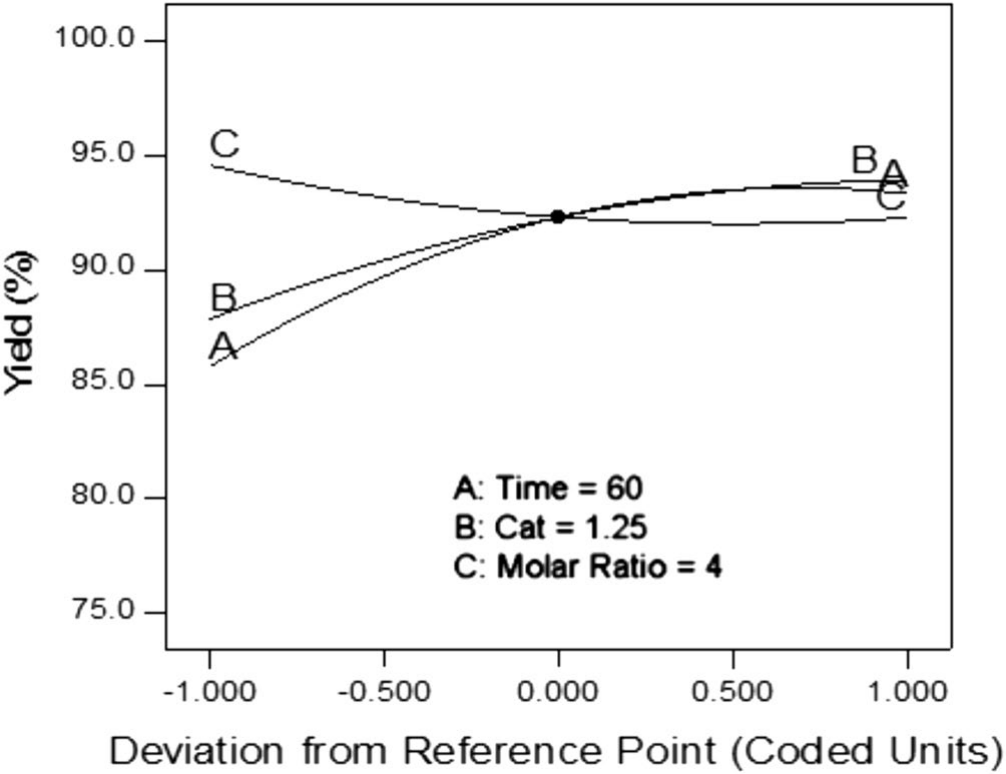
The main effect of independent variables on the bio-lubricant yield based on coded levels.

Which A, B and C are related to the coded levels of the independent variables of reaction time, potassium carbonate catalyst concentration (K_2_CO_3_) and the molar ratio of methyl ester to TMP, respectively. At a glance, the effect of each of the independent variables separately on the reaction efficiency was shown in Fig. 6. The effect of variables A and B on the yield in the present system was direct and by increasing their actual or coded values, the yield first increased and then remained constant. Variable C or the molar ratio had an adverse effect on the yield and with increasing the molar ratio, it first decreased and then remained constant. However, according to the ANOVA results in Table 4, the proposed regression model was statistically significant in predicting the bio-lubricant production yield.

**Table 4:** The ANOVA results.

Source	Sum of squares	df	Mean square	F-value	p-value
Model	279.59	9	31.07	37.63	< 0.0001
A-Time	113.16	1	113.16	137.06	< 0.0001
B-Catalyst	73.38	1	73.38	88.88	< 0.0001
C-Molar ratio	10.31	1	10.31	12.49	0.0095
AB	22.48	1	22.48	27.22	0.0012
AC	14.42	1	14.42	17.46	0.0041
BC	0.6696	1	0.6696	0.8110	0.3978
A^2^	31.62	1	31.62	38.30	0.0005
B^2^	8.40	1	8.40	10.18	0.0153
C^2^	5.23	1	5.23	6.34	0.0400
Residual	5.78	7	0.8256		
Lack of fit	2.98	3	0.9941	1.42	0.3602
Pure error	2.80	4	0.6993		
Cor total	285.37	16			

The Model F-value of 37.63 implies that the model is significant. There is only a 0.01% chance that an F-value this large could occur due to noise. The F-value of 1.42 for lack of fit, implies adequate model precision. In other words, there is a 36.02% chance that the fitted model could occur due to noise.

Based on the results of Table 4, the main effects of all variables, namely reaction time, catalyst concentration and molar ratio were statistically significant at 1% level. Also, the interaction effect of reaction time and catalyst concentration (AB) as well as time and molar ratio (AC) at 1% level was significant, but the interaction effect of catalyst concentration and molar ratio (BC) on the yield was not significant. The results of the analysis of variance showed that according to the values of the sum of squares and F-value, the most changes in the reaction yield were affected by the two main variables of reaction time and the amount of catalyst.

**Figure 7:**
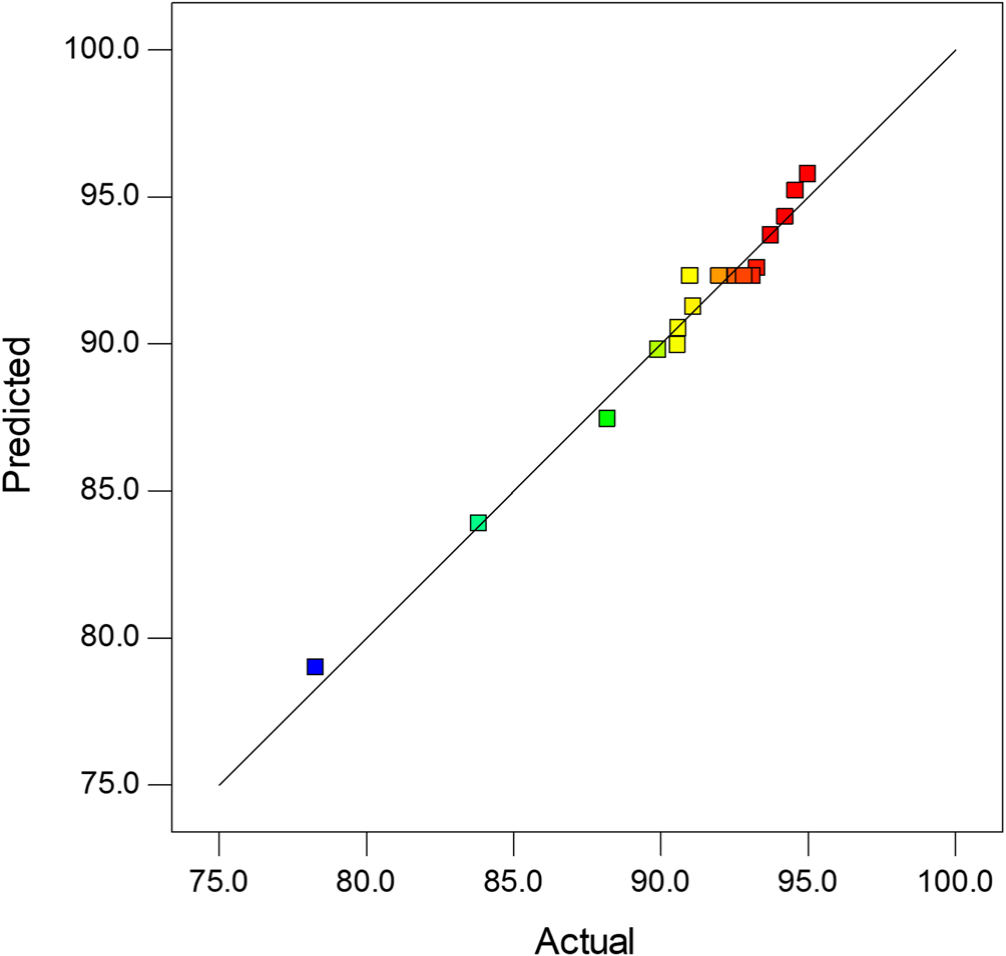
Actual values versus predicted values.

Figure 7 shows a comparison of the yield measured experimental values versus the yield values predicted by the regression model. As can be seen, there is a very good correlation between the actual data and the values predicted by the regression model (R^2^ = 0.9797).

### Effects of experimental factors

**Figure 8:**
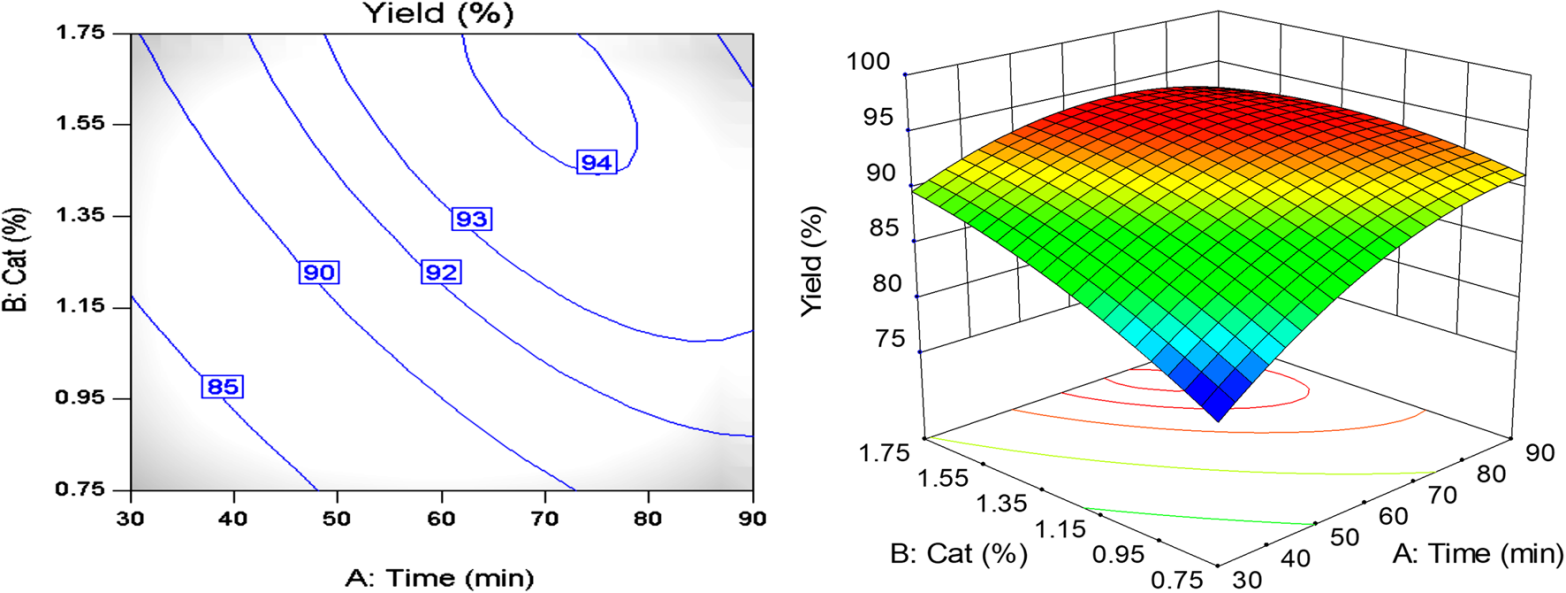
Interaction effect of reaction time and catalyst concentration on bio-lubricant yield.

The interaction effect of reaction time and catalyst concentration on the reaction yield is shown in the form of a 3D surface diagram and the contour plot in Fig. 8. According to this figure, the reaction yield increased with increasing time and after reaching its maximum value, it remained constant and even decreased (at the highest values of catalyst concentration and reaction time). At higher time levels, due to the decomposition of TMP ester molecules to methyl ester, the reaction was reversed and the reaction yield decreased^31^. By keeping the molar ratio constant at the level of 4, the reaction yield reached its maximum value of 94.14%. at this condition, the catalyst concentration and the time were set at 1.65% and 70 min respectively.

In another study, optimal conditions of the process for maximum yield (96.4%), were K_2_CO_3_ catalyst concentration of 1 wt%, the reaction time of 2 h, methyl ester to TMP molar ratio of 3.9, the temperature of 130 °C and vacuum pressure of 3.87 kPa^29^.

**Figure 9:**
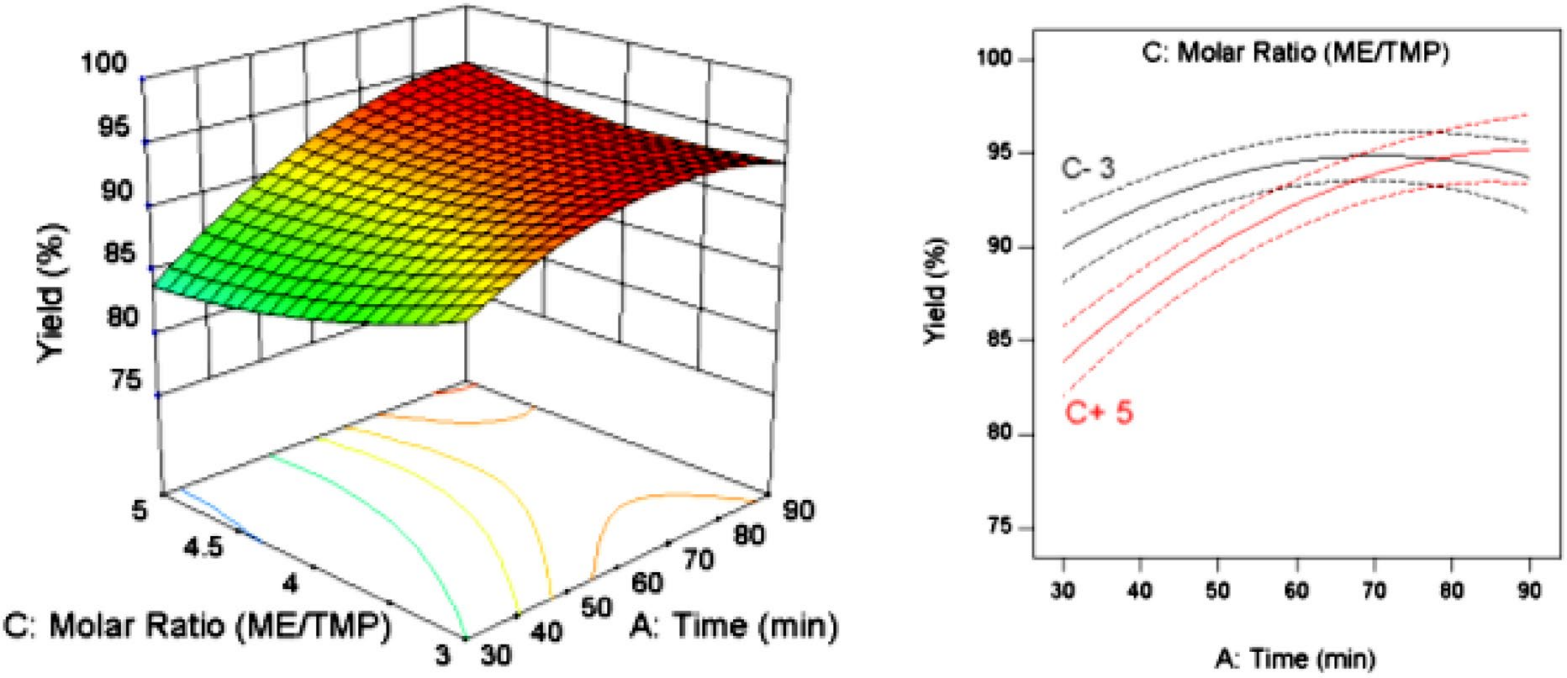
Interaction effect of reaction time and molar ratio.

Figure 9 shows the interaction effect of reaction time and molar ratio on the yield. As can be seen, increasing the time in different molar ratios increased the yield. Further increases in time kept this trend constant or led in the yield drop. Also, the reaction yield at lower molar ratios was higher than at elevated molar ratios. Although at low molar ratios the yield was higher in shorter reaction time, the trend increased with a gentle slope and after reaching its maximum in 60 min, it started to decline. At high molar ratios, the upward trend of the reaction yield continued with a steeper slope and remained almost constant during the reaction time of around 90 min. At this time, both curves corresponding to different molar ratios collided and intersected each other in approximately 70 min. It implies that to achieve maximum yield at higher molar ratios, the reaction time should be increased and at shorter reaction time, the molar ratio should be reduced. Also, by considering other effective conditions and parameters, optimal conditions of the variables can be obtained.

### Process optimization

**Figure 10:**
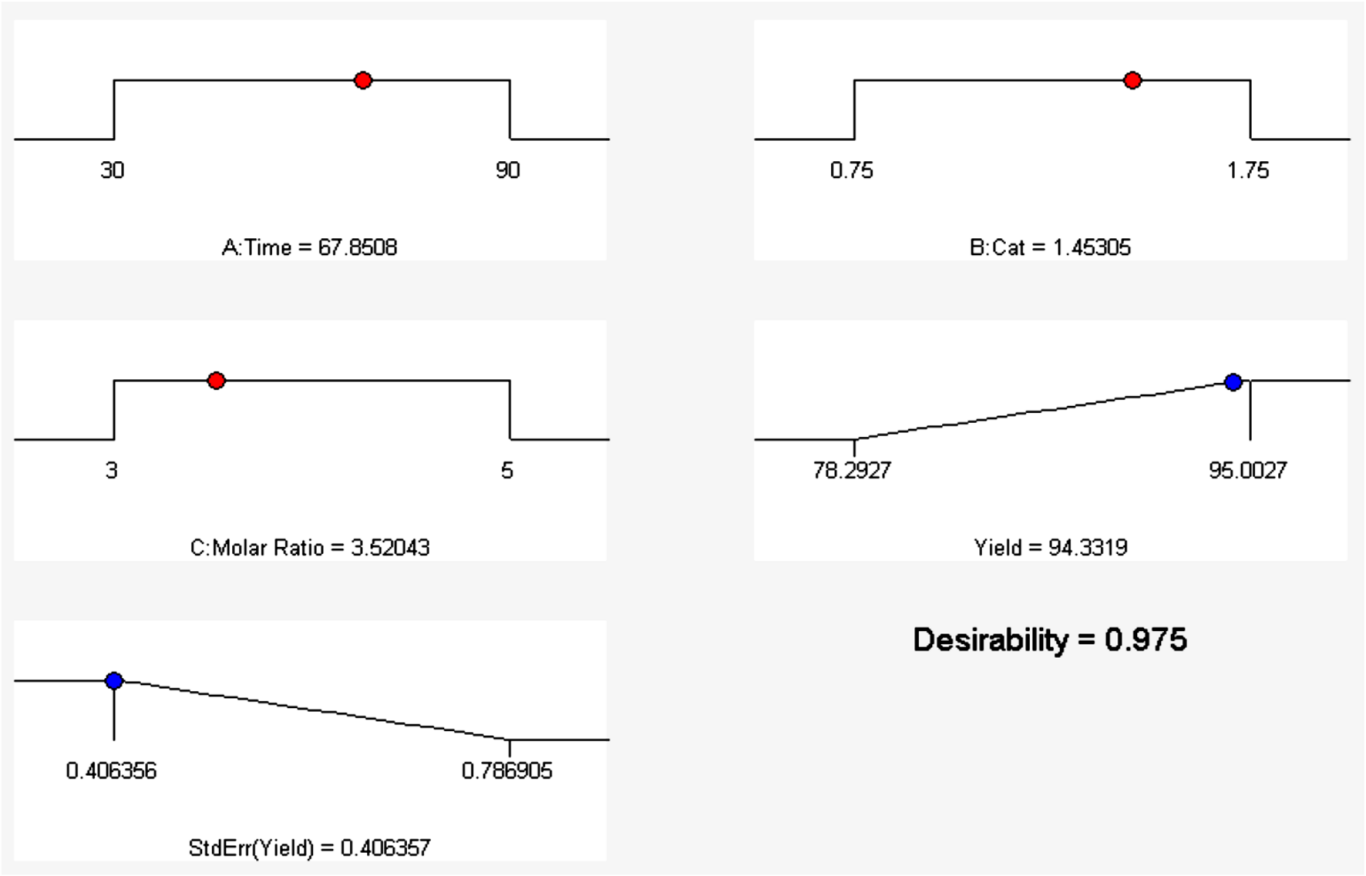
Optimization of parameters affecting the bio-lubricant reaction.

Optimization means obtaining the conditions under which the answer to the problem is directed towards the predetermined goal. The present optimization aimed to obtain the maximum reaction efficiency with the least standard deviation. Thereby in the optimization process, the importance of maximizing yield had a factor of 5 (highest coefficient) and the importance of standard error minimization had a factor of 3. Considering the above conditions and solving the optimization problem by Design-Expert software, an optimal combination was obtained with a time of 67.8 min, a catalyst concentration of 1.4 wt% and a molar ratio of 3.5. In this condition, the reaction yield will be 94.3% with desirability of 0.975 (Fig. 10). Of course, this combination of conditions is one of the solutions to the optimization problem that the software shows as the highest desirability and the first answer. Several other combinations in the same range could be offered with lower desirability as the answer to the optimization problem.

### Analysis of physicochemical properties

At the end of the experimental steps, a sample of product synthesized under the above-mentioned optimal condition was purification and its physicochemical properties were compared with the ISO-VG standard (Table 5). The results clearly showed that many of the obtained properties met the requirements of the standard. The pour point of the product (− 4) was a bit higher than the ISO-VG standard which can be attributed to the presence of unsaturated bonds in the fatty acids structure of camelina oil. However, in this case, adding a proper amount of pour point depressant additive is recommended.

**Table 5:** Characteristics of bio-lubricant produced from camelina methyl ester.

Properties	Unit	Values for produced bio-lubricant	Standard of ISO VG22	Standard of ISO VG10
Density (°C 15)	kg/m^3^	892	–	–
Kinematic viscosity (°C 100)	mm^2^/s	4.29	> 4.3	> 2.68
Kinematic viscosity (°C 40)	mm^2^/s	16.24	> 19.8	> 9
Viscosity index	–	188.16	107	104
Pour point	°C	− 4	− 6	− 6
Flash point	°C	172	> 93	> 101

## Conclusions

In the present study, the feasibility of using camelina oil, as a promising industrial crop was investigated for bio-lubricant (TMP ester) production. In this regard, process intensification of the mass-transfer-limited transesterification reaction of methyl ester obtained from camelina oil was conducted using microwave irradiations. The RSM-BBD approach was also applied for optimization of the experimental factors and to find the optimal levels at which the highest transesterification reaction yield is obtained. The time and molar ratio had a direct effect on the reaction efficiency and increasing their values was an increasing trend. The molar ratio had an inverse effect and with its increase, the reaction efficiency was reduced. Indeed, setting the microwave power at 800 W can greatly reduce the conventional reaction time (10 min instead of 120 min). Thereby, it can be concluded that microwave irradiation is an efficient method for process intensification of bio-lubricant production. Moreover, comparing the physicochemical properties of TMP ester produced from camelina oil with the requirements of ISO VG standard indicated that, camelina oil is an appropriate industrial crop for bio-lubricant production.

## Supplementary Information

Supplementary Information.

## Data Availability

All data generated or analyzed during this study are included in this published article (and its supplementary information files).
